# The German MultiCare-study: Patterns of multimorbidity in primary health care – protocol of a prospective cohort study

**DOI:** 10.1186/1472-6963-9-145

**Published:** 2009-08-11

**Authors:** Ingmar Schäfer, Heike Hansen, Gerhard Schön, Wolfgang Maier, Susanne Höfels, Attila Altiner, Angela Fuchs, Ferdinand M Gerlach, Juliana J Petersen, Jochen Gensichen, Sven Schulz, Steffi Riedel-Heller, Melanie Luppa, Siegfried Weyerer, Jochen Werle, Horst Bickel, Kerstin Barth, Hans-Helmut König, Anja Rudolph, Birgitt Wiese, Jana Prokein, Monika Bullinger, Olaf von dem Knesebeck, Marion Eisele, Hanna Kaduszkiewicz, Karl Wegscheider, Hendrik van den Bussche

**Affiliations:** 1 Department of Primary Medical Care, University Medical Center Hamburg-Eppendorf, Martinistr. 52, 20246 Hamburg, Germany; 2 Department of Medical Biometry and Epidemiology, University Medical Center Hamburg-Eppendorf, Martinistr. 52, 20246 Hamburg, Germany; 3 Department of Psychiatry and Psychotherapy, University of Bonn, Sigmund-Freud-Straße 25, 53105 Bonn, Germany; 4 Department of General Practice, University of Düsseldorf, Moorenstraße 5, 40225 Düsseldorf, Germany; 5 Institute for General Practice, University of Frankfurt am Main, Theodor-Stern-Kai 7, 60590 Frankfurt am Main, Germany; 6 Institute for General Practice, University of Jena, Bachstraße 18, 07743 Jena, Germany; 7 Public Health Research Unit, Department of Psychiatry, University of Leipzig, Semmelweisstr. 10, 04103 Leipzig, Germany; 8 Central Institute of Mental Health, J 5, 68159 Mannheim, Germany; 9 Department of Psychiatry, Technical University of Munich, Ismaninger Str. 22, 81675 München, Germany; 10 Health Economics Research Unit, Department of Psychiatry, University of Leipzig, Liebigstr. 26, 04103 Leipzig, Germany; 11 Institute for Biometry, Hannover Medical School, 30623 Hannover, Germany; 12 Department of Medical Psychology, University Medical Center Hamburg-Eppendorf, Martinistr. 52, 20246 Hamburg, Germany; 13 Department of Medical Sociology, University Medical Center Hamburg-Eppendorf, Martinistr. 52, 20246 Hamburg, Germany; 14 Department of Social Medicine, University of Leipzig, Philipp-Rosenthal-Str. 55, 04103 Leipzig, Germany

## Abstract

**Background:**

Multimorbidity is a highly frequent condition in older people, but well designed longitudinal studies on the impact of multimorbidity on patients and the health care system have been remarkably scarce in numbers until today. Little is known about the long term impact of multimorbidity on the patients' life expectancy, functional status and quality of life as well as health care utilization over time. As a consequence, there is little help for GPs in adjusting care for these patients, even though studies suggest that adhering to present clinical practice guidelines in the care of patients with multimorbidity may have adverse effects.

**Methods/Design:**

The study is designed as a multicentre prospective, observational cohort study of 3.050 patients aged 65 to 85 at baseline with at least three different diagnoses out of a list of 29 illnesses and syndromes. The patients will be recruited in approx. 120 to 150 GP surgeries in 8 study centres distributed across Germany. Information about the patients' morbidity will be collected mainly in GP interviews and from chart reviews. Functional status, resources/risk factors, health care utilization and additional morbidity data will be assessed in patient interviews, in which a multitude of well established standardized questionnaires and tests will be performed.

**Discussion:**

The main aim of the cohort study is to monitor the course of the illness process and to analyse for which reasons medical conditions are stable, deteriorating or only temporarily present. First, clusters of combinations of diseases/disorders (multimorbidity patterns) with a comparable impact (e.g. on quality of life and/or functional status) will be identified. Then the development of these clusters over time will be analysed, especially with regard to prognostic variables and the somatic, psychological and social consequences as well as the utilization of health care resources. The results will allow the development of an instrument for prediction of the deterioration of the illness process and point at possibilities of prevention. The practical consequences of the study results for primary care will be analysed in expert focus groups in order to develop strategies for the inclusion of the aspects of multimorbidity in primary care guidelines.

## Background

Multimorbidity is a highly frequent condition in older people that is supposed to significantly affect the patients' quality of life, functional status and life expectancy. But multimorbidity also is a complex phenomenon with an almost endless number of possible disease combinations of unclear implications. Therefore, it is not surprising that there is only marginal evidence on the causes and impact of multimorbidity today. To make things even more complex, there is a wide diversity in definitions and criteria of multimorbidity and there are many different measurement instruments and considerable differences in populations investigated [1].

Findings on the prevalence of multimorbidity are not consistent. A recent review by Marengoni of 33 population based studies published between 1989 and 2007 found prevalence rates in older people ranging from 21% to 98% [2]. Another review by Fortin et al. found rates of multimorbidity between 50 to 100% [3]. In both reviews, the ranges were due to differences in data sources, age groups investigated and the definitions of multimorbidity used.

In contrast to a larger number of cross-sectional epidemiological data, well designed longitudinal studies on the impact of multimorbidity on patients and the health care system have been remarkably scarce in numbers. The effect of multimorbidity was investigated in a review by Gijsen et al. [4]. According to these autors, multimorbidity was significantly associated with higher mortality, increased disability, a decline of functional status and a lower quality of life. Multimorbidity was also associated with an increase in health care utilization, i.e. number of physician encounters, rate of hospitalization, length of hospital stay, drug intake and costs [4,5]. Several studies also suggest an interrelation between mental and somatic disorders in multimorbidity clusters [6] and a protective effect of psychosocial resources of the patient [7].

There is a widespread consensus that actual health care delivery may not correspond to the needs of patients with multimorbidity. The central medical professional for the care and management of multiple chronic diseases is the GP. This is related to his broad expertise but also to the usually long-standing relationship to older patients. However, there is little help for the GP concerning the treatment of patients with multimorbidity. At present, clinical practice guidelines are mostly focussed on one disease only. Although adhering to current clinical practice guidelines in the treatment of multimorbidity may therefore even have adverse effects, the GP is widely left alone in adjusting the care for these patients [8]. In general, the diversity of results in studies on multimorbidity is not sufficient to provide robust evidence for the care of patients with multimorbidity [9].

In summary, the specific elements and processes in multimorbidity, the interactions and possible synergies of the diseases within multimorbidity clusters are basically a black box up to today. It remains unclear to which extent single disease combinations and to which extent a generalizable influence of multimorbidity contribute to the various outcomes [4]. Also, how multimorbidity develops over time is largely unknown.

The wide variety of possible disease combinations with diverse impacts on the patient makes it difficult to tackle the phenomenon of multimorbidity. In our approach, we will try to discern a limited number of groups of disease combinations ("multimorbidity patterns") with comparable impact (e.g. on quality of life and/or functional status). The statistical analyses will take the different multimorbidity patterns into account.

As a result of these considerations, the aims of the study are to:

• identify clusters of combinations of diseases/disorders (multimorbidity patterns) in the elderly general practice population and to determine their frequency and severity in relation to each other;

• investigate the development of these clusters over time (12 years), especially with regard to the internal interaction between the diseases within the cluster (addition, synergism, buffer, protection);

• analyse the relationship of mental and somatic disorders in these patterns;

• identify prognostic variables for the course of specific multimorbidity patterns;

• investigate the somatic, psychological and social consequences of multimorbidity (patterns) for the patient's quality of life and functional status; and

• describe the utilization of health care resources and the costs of care of multimorbidity (patterns).

## Methods/Design

### Design of the study

The study is designed as a multicentre, prospective, observational cohort study of 3.050 patients from general practice. The patients will be recruited from GP surgeries in 8 study centers distributed across Germany (Bonn, Düsseldorf, Frankfurt/Main, Hamburg, Jena, Leipzig, Mannheim and Munich). In each surgery, 50 eligible patients will be contacted and asked to participate in the study. All contacted patients who are willing to participate will be included in the study. As we estimate a rate of positive responses between 40 and 50%, approx. 120 to 150 GP surgeries have to be recruited consecutively. Each study center will recruit 435 patients with the exception of Frankfurt/Main and Jena, which will recruit 220 patients each. This equates to 18 GP surgeries with 24 patients each in every study center (9 GP surgeries in Frankfurt and Jena respectively).

The study centers began to recruit GPs in July 2008. Subsequently patient recruitment and data collection at baseline started in the same month. It is projected to perform a total of 9 waves of data collection by means of both GP and patient interviews. Each wave will take 15 months to be accomplished.

### Sample Size

Due to the investigation of multiple outcomes and the observational character of the study, there is no issue of statistical power to be considered. Nevertheless, we can derive from our experience with the similarly designed AgeCoDe-study that a sample size of 3.050 patients can be managed well in 8 study centres and will allow valid multivariate data analysis [10]. We expect a drop-out rate of 10% from baseline to first follow-up and a drop-out rate of 5% for all other consecutive waves.

### Inclusion of participants

Participating GPs will retrieve a list of all patients born between 1.7.1923 and 30.6.1943 who have consulted them within the last completed quarter (i.e. 3 month period). Out of all eligible patients from this list 50 patients will be selected at random (using random number tables) and invited to participate in the study by a letter from their GP. In case of interest, the patient will consult the GP and receive written and oral information. The information covers aims and procedures of the study, selection of participants, data collection, processing and storage as well as possibilities for cancellation. In case of acceptance, participants will have to sign an informed consent form to participate in the study. For each surgery, recourse and number of excluded patients per exclusion criteria will be documented. We will estimate the actual selection bias from age, gender and morbidity data.

Participants will be interviewed at all follow-ups by the same interviewer, if possible. This personal relationship is supposed to reduce the possibility of loss to follow-up. From follow-up 1 on, each participant will be asked to designate a contact person allowed to give information in the case the participant cannot be contacted. In case of drop out we will register the reason why (e.g. death, relocation, cancellation of participation in study etc.).

### Exclusion criteria

• Residence in a nursing home (inappropriateness for longitudinal studies because of an average life expectancy of 6 months after institutionalization).

• Severe illness probably lethal within three months according to the GP.

• Insufficient ability to speak and read German.

• Insufficient ability to consent (e.g. dementia).

• Insufficient ability to participate in interviews (e.g. blindness, deafness).

• Poorly known patients to the GP because of accidental consultation.

• Participation in other studies at the present time.

### Definition of multimorbidity

Multimorbidity is usually defined as the presence of two or more chronic diseases at the same time. Two chronic diseases are present in almost all people aged 65 years or older. In order to enrich the sample, we therefore decided to include only patients with at least three chronic conditions. Furthermore, single diseases are unequally distributed within the multimorbidity spectrum. For example, hypertension, hyperlipidemia and low back pain are present in between 50 and 75% of these age groups according to our (unpublished) analysis of the data of the Gmünder ErsatzKasse, a German statutory health insurance with 1.5 million members.

An unselected application of the three-disease-criterion would have resulted in an overrepresentation of these very frequent diseases in the study population. This would have led to little interest of the medical community in the results, as practical conclusions would have been limited to a very small number of patterns.

In order to ensure a wide range of diseases and syndromes, those with a prevalence of more than 25% were not used for inclusion into the sample. Nevertheless, these highly prevalent entities are frequently combined with the relatively lower prevalent ones and therefore still part of the sample. All diseases (including those not used for patient inclusion, e.g. hypertension) are registered in the morbidity spectrum of the patients and accounted for in the pattern analysis in order to obtain a complete picture of the diseases/syndromes in a patient.

### Disease list for inclusion (ICD10 Codes in brackets)

• alcohol abuse; alcoholic liver disease (F10;K70;K76);

• anaemia (D63-D64);

• anxiety disorders (F40-F41);

• arthrosis (excludes: osteoarthritis of spine) (M15-M19);

• atherosclerosis; intermittent claudication (I70;I73.9);

• cardiac arrhythmia (I44-I49);

• chronic ischemic heart disease; angina pectoris (I20;I25);

• chronic lower respiratory diseases (J40-J47);

• chronic stroke; transient cerebral ischemic attack; impaired cerebral blood flow (I60-I64;I67;I69;G45);

• chronic thyroid disorders; goitre (E01-E05;E06.1-E06.3;E06.5;E06.9;E07);

• depressive disorders (F32-F33);

• diabetes mellitus (E10-E14);

• disorders of vestibular function; dizziness and giddiness (H81;R42);

• diverticular intestinal disease (K57);

• hearing loss (H90-H91);

• heart failure (I50);

• malignant tumours (C00-C26;C30-C41;C43-C58;C60-C97;D00-D09;D37-D48);

• migraine (G43);

• neuropathies (G50-G64);

• non-rheumatic mitral valve or aortic valve disorders (I34-I35);

• osteoporosis (M80-M82);

• parkinson's disease (G20);

• psoriasis (L40);

• renal failure (N18-N19);

• rheumatoid arthritis; other soft tissue disorders (M05-M06;M79);

• somatoform disorders (F45);

• urinary incontinence (N39.3-N39.4;R32);

• varicose veins of lower extremities (I83);

• visual disturbances (H25-H26;H28;H33-H36;H40;H43;H47;H53-H54).

### Documentation of multimorbidity

The morbidity of the patients will be registered via chart review, GP-interviews and patient interviews.

### Chart review

For the last quarter, all ICD10 diagnoses from patients' charts in the GP documentation system will be retrieved for all recruited patients.

### GP interview

The GP will provide the disease spectrum of the patient by means of a standardized documentation instrument which covers:

1. all diseases described above (amended during follow-up data collection for chronic indications frequently mentioned in the open questions in previous waves);

2. neoplasms under 14 ICD10 subject headings;

3. mental and behavioral disorders under 11 ICD10 subject headings;

4. two open questions regarding further chronic and acute conditions not mentioned in the chronic diseases list.

For each illness, the GP will indicate duration (presence since how many years) and severity (regarding prognosis and subjective burden) on a Likert-type scale ranging from 0 = marginal to 4 = very severe.

### Patient interview

A similar list of diseases will be used in patient interviews, with the following modifications:

• due to the lack of comparison possibilities for the patient, the severity of the single diseases will not be rated by the patient;

• ICD-based psychic diseases are not included in the patient list. Assessment of the psychic situation of the patient from his/her view will be done with the following screening tests:

1. a culturally adapted version of the Four Dimensional Symptom Questionnaire (4DSQ) [11] for depression, anxiety, somatization and distress in primary care patients;

2. the Geriatric Depression Scale (GDS) [12];

3. the presence of cognitive disorders is assessed with the Clinical Dementia Rating (CDR) [13].

As a part of the patient interview a complete medication survey will be performed. The interviewer will collect data on all pharmaceutical products used by the patient within the last three months. The data include product name, pharmaceutical form, German national drug code ("Pharmazentralnummer" – PZN-), periodic or prn (pro re nata) medication, dosage and frequency (for periodic medication). The interviewer will ask the patient to show the packages of the pharmaceuticals to get the most valid information. If product name and drug code are not available, the patient will be asked for the medical indication of the drugs.

The total burden of morbidity will be measured by several multimorbidity indices, which will be automatically calculated from the disease data collected in the GP interviews and data on medication from patient interviews respectively:

• the count of the number of chronic conditions [14], without taking the disease severity in account (Unweighted Disease Count);

• the total number of chronic conditions weighted by disease severity as rated in GP interview (Weighted Disease Count);

• the diagnosis-related comorbidity score developed by Charlson et al. (Charlson Index) [15];

• the Cumulative Illness Rating Scale for Geriatrics (CIRS-G) [16], a multimorbidity index based on disease severity grouped at organ system levels;

• the medication-based chronic disease score developed by Von Korff et al. (Von Korff Index) [17].

The different indices will be compared regarding their predictive values.

The interviewers will also collect the following data about the attending GPs and GP surgeries:

• number of attending doctors in surgery;

• number of patients per doctor;

• location of surgery;

• age, gender and specialty of attending doctor;

• date of setting up of the surgery.

The patient interviews will contain an assessment of health care utilization. The questionnaire was developed on the basis of cost diaries used in earlier studies [18]. A random sample of 1/3 of the patients will be asked to give extensive information about the following data, whereas the other 2/3 will receive a short version:

• number of hospitalizations and of hospital days;

• number of contacts with GPs, specialists and dentists;

• prescriptions of therapeutic measures (e.g. physiotherapy);

• stage of nursing needs (according to the Statutory Nursing Insurance);

• utilization of nursing services.

### Variables under study

The variables under study belong to four groups: morbidity (as described above), functional status, resources/risk factors and socio-demographic data. The domain of functional status includes activities and instrumental activities of daily living, motor skills, senses (i.e. hearing and vision), cognition, pain and health-related quality of life. Resources include physical activity, balanced nutrition, social support, general self-efficacy, utilization of medical services and the quality of medical care according to the Chronic Care Model [19]. Risk factors include physical inactivity, malnutrition, alcohol abuse, smoking, body-mass-index and waist-to-hip-ratio. Socio-demographic variables include age, gender, migrant status, marital status, living conditions, household size, education, former occupation, income and wealth.

Table 1 gives an overview of the variable groups, and the measuring instruments and data sources for the variables. The instruments were chosen according to comprehensiveness, established reliability and validity, appropriateness for the age group ≥ 65 years, understandability and ease of administration in face-to-face interviews.

**Table 1:** Variables, measuring instruments and data sources

Morbidity	Measuring instrument	Data source
Diagnoses	ICD-10 codes	chart review
Diseases	self-developed questionnaire	GP interview
	self-developed questionnaire	patient interview
	Four-Dimensional Symptom Questionnaire (4DSQ) [11]	patient interview
	Geriatric Depression Scale (GDS) [12]	patient interview
	Clinical Dementia Rating (CDR) [13]	rated by interviewer
**Functional Status**		

Activities of daily living	Barthel Index [22]	patient interview
	Instrumental Activities of Daily Living (IADL) Scale [23]	patient interview
Motor skills	FFB-Mot [24]	patient interview
Vision and hearing	2 items rated on a 4 point scale	patient interview
Cognitive impairment	CERAD [25] subtests word list and animal naming	cognitive tests by interviewer
	Letter Digit Substitution Test (LDST) [26]	cognitive tests by interviewer
Pain	Graded Chronic Pain Scale (GCPS) [27]	patient interview
Health-related quality of life	EuroQoL (EQ-5D) Scale [28]	patient interview
**Resources/Risk Factors**		

Physical risk factors	Body-Mass-Index and Waist-to-Hip-Ratio	physical checkup by GP
Physical activity	International Physical Activities Questionnaire (IPAQ) [29]	patient interview
Nutrition	12 items for frequency and portion size of nutriments	patient interview
Alcohol use	AUDIT-C [30]	patient interview
	total amount of alcohol consumption per week	patient interview
Smoking behavior	9 items indicating current smoking status and pack years	patient interview
General self-efficacy	General Self-efficacy Scale [31]	patient interview
Social support	F-SOZU K14 [32]	patient interview
Medical care	Patient Assessment of Chronic Illness Care (PACIC) [33]	patient interview at baseline
	age and gender of GP/size and location of surgery	GP interview
Utilization of medical services (incl. medication)	15 items (short form), respectively 91 items (long form)	patient interview
**Sociodemographic Data**		

Age and gender	age and gender	chart review
Migrant status	standardised indicators for mapping migrant status [34]	patient interview at baseline
Marital status	marital status	patient interview
Living conditions	independent or assisted living	patient interview
Household size	household size/household members under 15 years	patient interview
Education	CASMIN classification [35]	patient interview at baseline
Former occupation	German epidemiological standard questionnaire [36]	patient interview at baseline
Income	household size adjusted net income	patient interview
Wealth	home ownership	patient interview

Visual aids with possible response options will guide interviewees in the different standardized questionnaires. For each interviewer, age, gender, experience, and professional background will be documented to assess (and statistically control for) interviewer effects.

### Quality assurance

Procedures for prevention of insufficient data quality, detection of inaccurate or incomplete data and actions to improve data quality will be performed, e.g. user reliability trainings, automatic plausibility and integrity checks and data error reports to the collaborating centers. The centers will receive feedback by quality reports for which the indicators of the national guidelines of the TMF-project ("telematics platform for medical research networks") for data quality will be applied. These quality controls will be conducted by the Institute for Biometry of Hannover Medical School. For every survey wave, the Institute will conduct a source data validation of a 1% random sample of all questionnaires to calculate input data errors.

### Data security

The interviews will be performed by trained scientists and study nurses at the patients' homes and for the GPs in their surgeries using printed forms. Regular training sessions will be performed twice each year. An email list server will be used for clarification of not anticipated assessment problems. Survey sheets and patient contact sheets will be stored in separate lockers in the study centers. Data will be entered in the local centers via an internet based remote data entry system. The data are transferred via 128 bit SSL encryption. The data will be stored in a central database in the Institute for Biometry of Hannover Medical School. The access to the internal database and web server is controlled by two consecutive firewall systems. A pseudonym will be automatically created when the identification data are entered and a printed copy with the pseudonym and the identification data will be archived by an officiating notary ("data trustee"). The identification data will neither be electronically transferred nor stored. The members of the study group will have access to the electronic data entry system according a detailed concept of roles and rights. An audit trail will ensure an automatic protocol of all data entries, changes and deletions.

### Description of risks

It is not expected that participation in the study will expose the patients to any risks. Nevertheless, monitoring of the impact of the study on patients will be performed by the interviewers and counseling given in case participants experience any discomfort or harm.

### Ethics approval

The study is conducted in compliance with the Helsinki Declaration. The study protocol was approved by the Ethics Committee of the Medical Association of Hamburg in February 2008 and amended in November 2008 (Approval-No. 2881).

### Data Analysis

#### Cross-sectional analysis

Recruitment and baseline data will be used to develop statistical models that relate the independent variables to the dependents:

• Multimorbidity patterns of similar severity will be identified by methods of supervised learning (methods of recursive partitioning like CART [Classification and Regression Trees], logistic regression).

• We will conduct different regression modelling strategies [20]. The basic model for each dependent will contain the independents as regressors, and interactions between them.

• Mixed models will be applied allowing to take the GP-induced cluster structure into account.

• The correspondence of GP-rated and patient-rated data will be analysed using inter-rater-reliability (Cohen's kappa).

#### Longitudinal analysis

• Based on the results of the cross-sectional analysis, hypotheses will be formulated as far as the individual outcomes (dependents) at the end of the follow-up period are concerned. Predictive scores will be calculated [21].

• As soon as the follow-up data are available, mixed models which include the baseline variables will be fitted for the follow-up data or the changes from baseline, respectively.

• Structural equation modeling will be applied to map causal chains between dependents.

• A classification system will be derived which groups patients as they are comparable with respect to intensity of care and costs.

## Discussion

The project will be the first large scale and longitudinal investigation of multimorbidity in Germany based on a cohort of multimorbid patients randomly selected from general practice data bases. The project will help to discern distinct multimorbidity patterns and identify variables associated with these patterns. A better understanding of the individual variability in the process of multimorbidity is necessary for a better quality of medical care, better support of patients' self management and a more effective and efficient allocation of resources.

Therefore, the main aim of the cohort study is to monitor the course of the illness process and to analyse for which reasons medical conditions are stable, deteriorating or only temporarily present. The results will allow the development of an instrument for prediction of the deterioration of the illness process and point at possibilities of prevention.

The practical consequences of the study results for primary care will be analysed in expert focus groups of GPs and nurses, supplemented by further experts (e.g. geriatricians). The aim is to develop strategies for the inclusion of the aspects of multimorbidity in primary care guidelines. This will include:

• rating of existing guidelines with regard to their (in)appropriateness for primary care;

• prioritization of multimorbidity patterns according to the needs for specific guidelines;

• needs for special services for patients with multimorbidity (e.g. improvement of the existing education programs; possibilities and limits of multimorbidity oriented disease management programs).

## Competing interests

The authors declare that they have no competing interests.

## Authors' contributions

HvdB, IS, HK, ME, KW and BW conceived the study. AA, KB, HB, MB, AF, JG, FMG, HH, SH, HHK, ML, WM, JJP, JP, SRH, AR, GS, SS, OvdK, JW and SW participated in implementing the study. IS, HvdB and HH drafted the manuscript. All authors commented on the draft and approved the final version of the manuscript.

## Pre-publication history

The pre-publication history for this paper can be accessed here:

http://www.biomedcentral.com/1472-6963/9/145/prepub
